# Complete Genome Sequence of *Dehalobacterium formicoaceticum* Strain DMC, a Strictly Anaerobic Dichloromethane-Degrading Bacterium

**DOI:** 10.1128/genomeA.00897-17

**Published:** 2017-09-14

**Authors:** Gao Chen, Robert W. Murdoch, E. Erin Mack, Edward S. Seger, Frank E. Löffler

**Affiliations:** a Center for Environmental Biotechnology, University of Tennessee, Knoxville, Tennessee, USA; b Department of Civil and Environmental Engineering, University of Tennessee, Knoxville, Tennessee, USA; c Department of Microbiology, University of Tennessee, Knoxville, Tennessee, USA; d Department of Biosystems Engineering and Soil Science, University of Tennessee, Knoxville, Tennessee, USA; e Biosciences Division, Oak Ridge National Laboratory, Oak Ridge, Tennessee, USA; f Corporate Remediation Group, E. I. DuPont de Nemours and Company, Newark, Delaware, USA; g The Chemours Company, Wilmington, Delaware, USA

## Abstract

*Dehalobacterium formicoaceticum* utilizes dichloromethane as the sole energy source in defined anoxic bicarbonate-buffered mineral salt medium. The products are formate, acetate, inorganic chloride, and biomass. The bacterium’s genome was sequenced using PacBio, assembled, and annotated. The complete genome consists of one 3.77-Mb circular chromosome harboring 3,935 predicted protein-encoding genes.

## GENOME ANNOUNCEMENT

Dichloromethane (DCM) is both naturally occurring (1) and synthesized by industry. Whereas aerobic DCM degradation has been studied in detail (2–4), DCM degradation under anoxic conditions is unclear (5–9). *Dehalobacterium formicoaceticum* is the only published isolate utilizing DCM as the sole energy source under anoxic conditions (7). *D. formicoaceticum* is a strictly anaerobic Gram-positive rod-shaped spore-forming bacterium affiliated with the *Peptococcaceae* family (7, 10). Previous physiological and biochemical studies suggested that DCM metabolism involves initial dechlorination and the formation of methylene tetrahydrofolate, which is funneled into the Wood-Ljungdahl pathway (11). The draft genome of “*Candidatus* Dichloromethanomonas elyunquensis,” identified as the DCM degrader in an anaerobic consortium, has been published (12), and here we report genomic information for axenic *D. formicoaceticum*.

*D. formicoaceticum* was obtained from the American Type Culture Collection (ATCC 700118) and cultivated in defined anoxic bicarbonate-buffered mineral salt medium (13, 14) containing DCM as the sole energy source. Genomic DNA was isolated using the cetyltrimethylammonium bromide method (15). The long-insert library for sequencing on the RS II platform (Pacific Biosciences, Menlo Park, CA, USA) was prepared by shearing DNA with a g-TUBE (Covaris, Woburn, MA, USA), targeting an average fragment size of 20 kb. The SMRTbell template preparation kit (Pacific Biosciences) was used to ligate hair-pin adapters to the fragmented DNA. The final library was size selected (BluePippin, Sage Science, Beverly, MA, USA) and sequenced on a single SMRT cell using PacBio P6-C4 chemistry with one 240-min movie. PacBio raw data were error corrected and assembled using the HGAP (SMRT Analysis version 2.3.0), Canu version 1.2 (16), and Celera version 8.2 assemblers with default parameters for bacterial genome assembly. The resulting assemblies were assessed for inconsistencies and misassembly using NUCmer version 3.0 whole-genome alignments (17) and Circleator plots (GC-skew) (18). Canu version 1.2 (16) generated a single contig representing the chromosome, which was polished using Quiver (SMRT Analysis version 2.3.0) to generate the final consensus genome sequence. The IGS prokaryotic annotation pipeline was used for coding gene prediction and functional annotation (19). The output annotations were loaded into a MySQL Chado relational database and accessed through the visualization tool Manatee (http://manatee.sourceforge.net). Protein-coding genes were predicted using Glimmer version 3 (20), and noncoding RNA genes were predicted using tRNAscan-SE (21) and RNAmmer version 1.2 (22).

The complete genome of *D. formicoaceticum* comprises one circular chromosome (3,766,545 bp) with an overall G+C content of 43.17%. A total of 3,935 predicted protein-encoding genes were identified in the genome. In total, 55 tRNAs and 17 rRNAs were identified, including six 5S rRNAs, five 16S rRNAs, and six 23S rRNAs. The genome harbors genes encoding all enzymes involved in the Wood-Ljungdahl pathway with a featured core acetyl coenzyme A synthase (*acs*) gene cluster (23). Genes encoding *c*-type cytochromes (7 genes), a complete NADH:ubiquinone oxidoreductase (Nuo) complex (11 genes), and one F_1_F_0_-ATPase (11 genes) were identified, suggesting chemiosmotic energy conservation. A complete set of sporulation genes is consistent with microscopic observations of spores (7). Genes encoding reductive dehalogenases were not found.

### Accession number(s).

The complete genome sequence of *D. formicoaceticum* has been deposited in GenBank under accession no. CP022121.
